# A comparison of hyperelastic constitutive models applicable to brain and fat tissues

**DOI:** 10.1098/rsif.2015.0486

**Published:** 2015-09-06

**Authors:** L. Angela Mihai, LiKang Chin, Paul A. Janmey, Alain Goriely

**Affiliations:** 1 School of Mathematics, Cardiff University, Senghennydd Road, Cardiff CF24 4AG, UK; 2 Institute for Medicine and Engineering, University of Pennsylvania, 3340 Smith Walk, Philadelphia, PA, USA; 3 Departments of Physiology and Physics and Astronomy, Institute for Medicine and Engineering, University of Pennsylvania, 3340 Smith Walk, Philadelphia, PA, USA; 4 Mathematical Institute, University of Oxford, Woodstock Road, Oxford OX2 6GG, UK

**Keywords:** constitutive models, elastic moduli, large strain, brain tissue, brain tumours, fat tissue

## Abstract

In some soft biological structures such as brain and fat tissues, strong experimental evidence suggests that the shear modulus increases significantly under increasing compressive strain, but not under tensile strain, whereas the apparent Young's elastic modulus increases or remains almost constant when compressive strain increases. These tissues also exhibit a predominantly isotropic, incompressible behaviour. Our aim is to capture these seemingly contradictory mechanical behaviours, both qualitatively and quantitatively, within the framework of finite elasticity, by modelling a soft tissue as a homogeneous, isotropic, incompressible, hyperelastic material and comparing our results with available experimental data. Our analysis reveals that the Fung and Gent models, which are typically used to model soft tissues, are inadequate for the modelling of brain or fat under combined stretch and shear, and so are the classical neo-Hookean and Mooney–Rivlin models used for elastomers. However, a subclass of Ogden hyperelastic models are found to be in excellent agreement with the experiments. Our findings provide explicit models suitable for integration in large-scale finite-element computations.

## 1. Introduction

Obtaining reliable constitutive models for the behaviour of tissues under loads is of the utmost importance when studying the response and evolution of organs in physiological and pathological conditions. For instance, the computational analysis of traumatic brain injury owing to shocks or blast waves in sports, combat or accidents relies on large finite-element codes based on the constitutive properties of brain tissues. Similarly, an understanding of how brain tumours change the mechanical and neurological environment during growth depends on the mechanical responses of both healthy tissue and tumours [1]. The response of adipose tissue to external loads is also a growing area of interest in clinical research, for example in treating patients with impaired mobility and in the pharmaceutical industry, particularly for the design of needle-free drug-delivery systems [2].

Recent experimental evidence [3–5] shows that soft biological tissues such as brain, gliomas, liver and fat have some unusual mechanical properties under loads, namely

(i) the shear modulus increases sharply as compression in the direction orthogonal to the shear direction increases;
(ii) the shear modulus remains almost constant or may decrease as tension in the direction orthogonal to the shear direction increases; and
(iii) the elastic modulus increases or remains almost constant when compression increases.

In particular, the shear modulus of normal brain can be increased nearly four times by compressive stresses. In addition, although at low strains, glioma brain tumours have similar elastic moduli to normal brain tissue (unlike other tumour types arising in breast tissue, for example [6]), at large strains, glioma tissue stiffens more strongly under compression than normal brain. However, while the shear modulus increases significantly when axial strain increases, the elastic modulus increases only slightly or not at all under increasing axial strain. During experimental tests, tissue samples exhibited a predominantly isotropic behaviour and their volume was reported to remain virtually constant.

**Figure 1.**
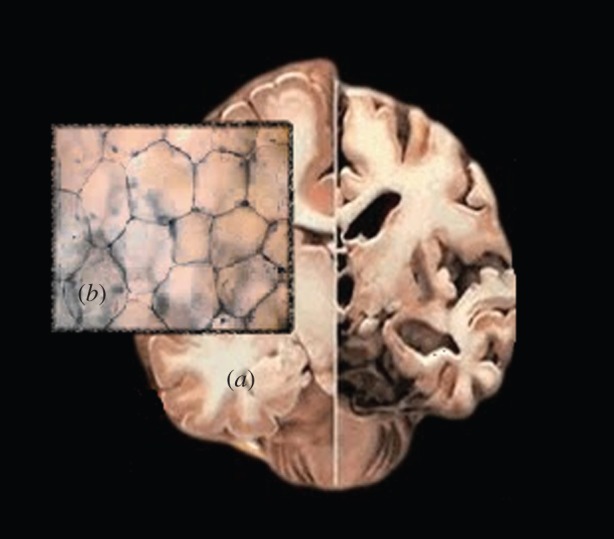
Graphical illustration of (*a*) brain and (*b*) fat tissues.

Our aim is to capture these seemingly contradictory mechanical behaviours, both qualitatively (theoretically) and quantitatively (numerically), within the framework of finite elasticity, by modelling a soft tissue as a homogeneous, isotropic, incompressible, hyperelastic material. First, we demonstrate analytically that, in large strain deformations, conditions (i)–(iii) can be satisfied simultaneously by Mooney–Rivlin models, but not by the neo-Hookean, Fung and Gent models. The neo-Hookean model can be derived from first principles and is suitable for materials with entropic elasticity and a Gaussian distribution of chains with quadratic strain energy. While the neo-Hookean model can be seen as a general second-order approximation of a strain–energy density, the Mooney–Rivlin model for incompressible systems is its third-order approximation and is known to be better suited than the neo-Hookean model to describe shear deformations in elastomers [7,8]. The Fung model was developed initially to capture the response of tissues with a high content of collagen fibres, such as skin and arterial walls [9,10]. This model exhibits a typical dramatic strain-stiffening response in uniaxial loading characterizing the extension of stiff crimpled collagen fibres. The Gent model further penalizes this extension by limiting the strain to a finite value, similar to worm-like chain models used in polymer physics [11]. As such, both the Gent and Fung models are suitable for tissues that derive their elasticity from a mixture of a soft elastic matrix and stiff fibres. However, aggregates of cells found in brain and in fat tissues are approximately equiaxed in structure with a large lipid content, and this accounts for their almost isotropic, incompressible properties, which likely originate from their cellular structure rather than fibres [4,12–15] (figure 1 and appendix A).

In this study, we provide a set of model examples for which we compare the values of the shear modulus under increasing compression or tension with experimental data for brain and fat tissues. The viscoelasticity of brain and adipose tissues was measured following the protocol described in [4]. The dynamic shear storage modulus *G*′ was measured as a function of time for increasing tensile or compressive strain (from 0% to 40%). Details are given in appendix A.

A hyperelastic constitutive material has a unique stress–strain relationship, independent of strain rate. However, the stress–strain response for viscoelastic materials changes with strain rate, and a strain–energy density function does not exist for these materials. Nonetheless, for many soft tissues, the shape of the nonlinear stress–strain curve is typically invariant with respect to strain rate. In this case, at fixed strain rate, the shear modulus may be captured by a nonlinear hyperelastic model (an example of this approach for fat tissues can be found in [16]).

The usual practice in constitutive modelling is to fit uniaxial data obtained under controlled compression, and less commonly under tension, with standard material models. This is due to the general experimental (and partly analytical) limitations to carry out proper assessment of stresses and deformations in multiple loading situations. This approach has been particularly successful for tissues that operate mostly under axial loading conditions, such as tendons or ligaments. However, soft tissues such as fat or brain, operate in highly varying and complex loading environments and exhibit responses that cannot be easily modelled by such an approach. The elasticity of these materials can be probed by subjecting samples to multiple loadings and, indeed, our study of the shear modulus under combined stretch and shear demonstrates that different constitutive models behave very differently under combined deformation, even though some of them may respond very similarly in axial deformation alone.

From our numerical results, we infer that the shear modulus for the Mooney material is too small compared with the experimental values at similar strains, but appropriate Ogden models are found which are in excellent agreement with the experiments, and thus conditions (i)–(ii) are satisfied by the corresponding shear modulus. The newly identified models are robust and suitable for use in large-scale finite-element computations. Furthermore, for the Mooney, Fung, Gent and Ogden models analysed here, the elastic modulus increases or remains almost constant as compression increases, and therefore, condition (iii) is satisfied numerically, whereas for the neo-Hookean material, this modulus decreases under increasing compression.

For the hyperelastic models under consideration, the associated strain energy functions and their ability to satisfy the conditions (i)–(iii), either theoretically or numerically, are summarized in table 1. The numerical results are shown at a glance in figures 3 and 4.

**Table 1.** Hyperelastic material models and their mechanical behaviour.

material model	strain energy function 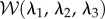	conditions (i)–(iii) satisfied (√)/failed (×)
neo-Hookean [17]	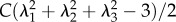 *C* independent of deformation	(i)×; (ii) √; (iii) ×
Mooney–Rivlin [18]	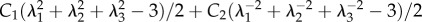 *C*_1_, *C*_2_ independent of deformation	(i) √; (ii) √; (iii) √
Fung [19]	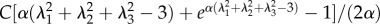 *C*, *α* independent of deformation	(i) √; (ii) ×; (iii) √
Gent [20]	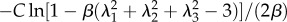 *C*, *β* independent of deformation	(i) √; (ii) ×; (iii) √
Ogden*_N_* [21]	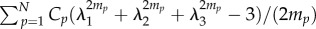 *C_p_*, *m_p_* independent of deformation	(i) √; (ii) √; (iii) √

## 2. Nonlinear elastic modulus and shear modulus relations

The homogeneous (affine) deformations analysed here are universal and controllable in the sense that they can be maintained in every homogeneous, incompressible, isotropic, elastic material by application of suitable surface tractions [22–26]. If the material is described by a strain energy function 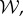 the associated Cauchy (true) stress has the Rivlin–Ericksen representation
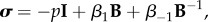
where *p* is the arbitrary hydrostatic pressure, **B** is the left Cauchy–Green strain tensor with the principal invariants *I*_1_, *I*_2_, *I*_3_ and
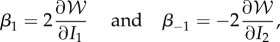
are the material response coefficients. Equivalently, in terms of the principal stretches *λ*_1_, *λ*_2_, *λ*_3_
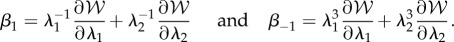


Henceforth, we assume that these material responses are consistent with the Baker–Ericksen (BE) inequalities stating that *the greater principal stress occurs in the direction of the greater principal stretch*, and the pressure–compression (PC) inequalities stating that *each principal stress is a pressure or a tension according as the corresponding principal stretch is a contraction or an elongation* [27–29].

### 2.1. The elastic modulus in finite tension or compression

We first consider a unit cube of incompressible hyperelastic material subject to the uniaxial tension or compression in the second direction2.1
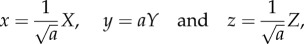
where (*x*, *y*, *z*) and (*X*, *Y*, *Z*) are the Cartesian coordinates for the current and the reference configuration, respectively, and *a* > 1 (tension) or 0 < *a* < 1 (compression) is constant.

For the deformation (2.1), the left Cauchy–Green strain tensor takes the form
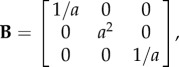
and the non-zero components of the associated Cauchy stress are
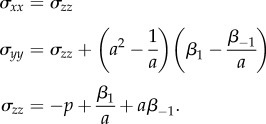
We define the *nonlinear elastic modulus* in the second direction as the ratio between the Cauchy (true) stress *σ_yy_* and the logarithmic (true) strain (the sum of all the small strain increments) 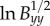 [24, p. 118]2.2
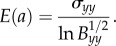


If *σ_xx_* = *σ_zz_* = 0, then (2.2) takes the form2.3
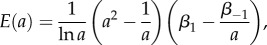
and if *σ_xx_* = *σ_zz_* ≠ 0, then by the PC inequalities, *σ_zz_* < 0 when 1/*a* < 1, and *σ_zz_* > 0 when 1/*a* > 1, hence2.4
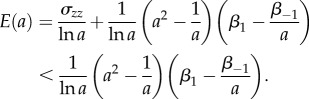


### 2.2. The shear modulus for finite shear superposed on axial stretch

**Figure 2.**
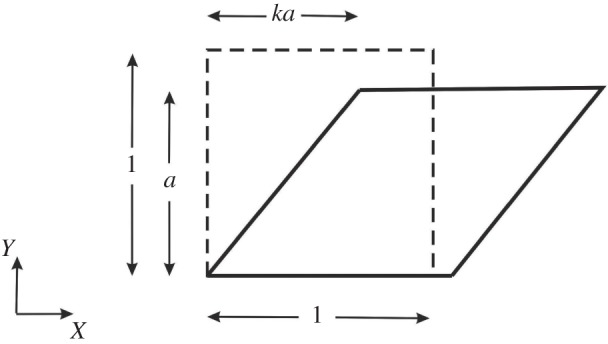
Schematic of cross section of unit cube (dashed line) deformed by combined stretch and shear (solid line).

We further examine a unit cube material sample deformed by the combined stretch and shear2.5
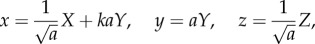
where (*x*, *y*, *z*) and (*X*, *Y*, *Z*) are the Cartesian coordinates for the deformed and the reference configuration, respectively, and *a* and *k* are positive constants representing the axial stretch and the shear parameter, respectively (figure 2).

For the deformation (2.5), the left Cauchy–Green strain tensor takes the form
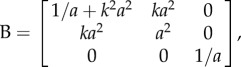
and the non-zero components of the associated Cauchy stress are
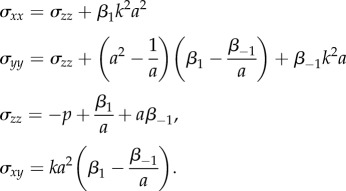
For the deformed cube, the shear strain on the inclined faces and the associated shear traction are, respectively
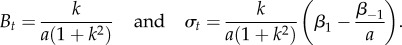


We define the *nonlinear shear modulus* as the ratio between the shear traction *σ_t_* and the logarithmic shear strain ln(*B_t_* + 1), i.e.2.6
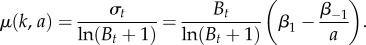


Then, the shear modulus (2.6) is independent of the hydrostatic pressure –*p* and is positive if and only if *β*_1_ − *β*_−1_/*a* > 0.

When the shear strain is small, the shear modulus (2.6) takes the form2.7
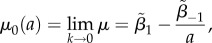
where
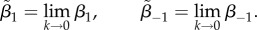


Assuming that *σ_zz_* = 0, we also define
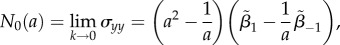
and obtain2.8
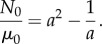
Therefore, as the axial stretch *a* increases, the magnitude of the normal force *N*_0_ relative to the shear modulus *μ*_0_ also increases. This is a *universal relation* [30], which holds independently of the material responses *β*_1_ and *β*_−1_, and is analogous to *Rivlin's formula* for a cylinder deformed by combined stretch torsion [26, p. 192]. Then, by (2.3) and (2.8)2.9
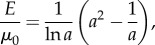
i.e. the ratio between the elastic modulus *E* and the shear modulus *μ*_0_ is also independent of the material parameters, and *E*/*μ*_0_ → 3 as *a* → 1.

If *σ*_zz_ ≠ 0, then by the PC inequalities, *σ_zz_* < 0 when 1/*a* < 1, and *σ_zz_* > 0 when 1/*a* > 1. In this case
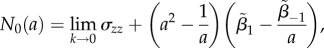
hence
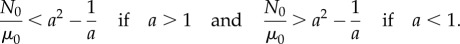
Then, by (2.4)
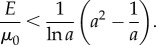


### 2.3. The behaviour of nonlinear hyperelastic models

For the hyperelastic materials listed in table 1, we examine the elastic modulus (2.3) and the shear modulus (2.7) as the magnitude of the compressive or tensile strain *b* = ln *a* increases. In view of the subsequent comparison with experimental data, we restrict our attention to the case when 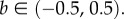

— For the neo-Hookean model, the shear modulus (2.7) is equal to
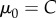
and is independent of strain. *Hence, condition* (*ii*) *is satisfied, but not* (*i*).
  Applying (2.9) the corresponding elastic modulus takes the form2.10
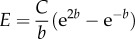
and increases as 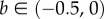 increases. *Thus, condition* (*iii*) *is not satisfied*.
— For the Mooney–Rivlin model, the shear modulus takes the form
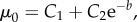
and, if *C*_1_ > 0 and *C*_2_ > 0, then this modulus decreases as *b* increases. *Hence, conditions* (*i*) *and* (*ii*) *are both valid*.
— By (2.9), the elastic modulus is equal to2.11
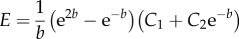
and, if 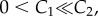 then this modulus decreases as 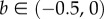 increases. *Thus, condition* (*iii*) *is also valid*.
— For the Fung model:
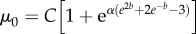
and, if *C* > 0 and *α* > 0, then this modulus decreases as 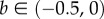 increases and increases as 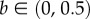 increases. *Hence, condition* (*i*) *is satisfied, but not* (*ii*).
  For this model, by (2.9), the elastic modulus is2.12
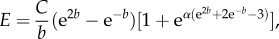
and there exists 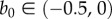, such that this modulus decreases as 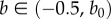 increases and increases as 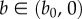 increases. *Thus, condition* (*iii*) *is not satisfied*.
— Similarly, for the Gent model:
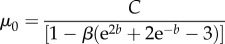
and, if *C* > 0 and *β* > 0, then this modulus decreases as 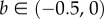 increases and increases as 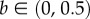 increases. *Hence, condition* (*i*) *is satisfied, but not* (*ii*).
By (2.9), the corresponding elastic modulus is2.13
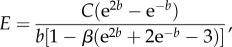
and there exists 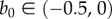, such that this modulus decreases as 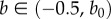 increases and increases as 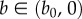 increases. *Hence, condition* (*iii*) *is not satisfied*.
— For the Ogden model:
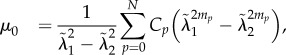
where
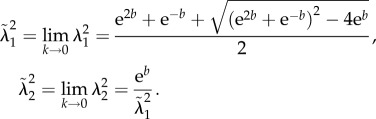
For this model, a general conclusion about the monotonicity of the shear modulus cannot be drawn, and particular cases need to be examined individually. We do this in §3 where hyperelastic models are treated numerically.

## 3. Numerical results

**Figure 3.**
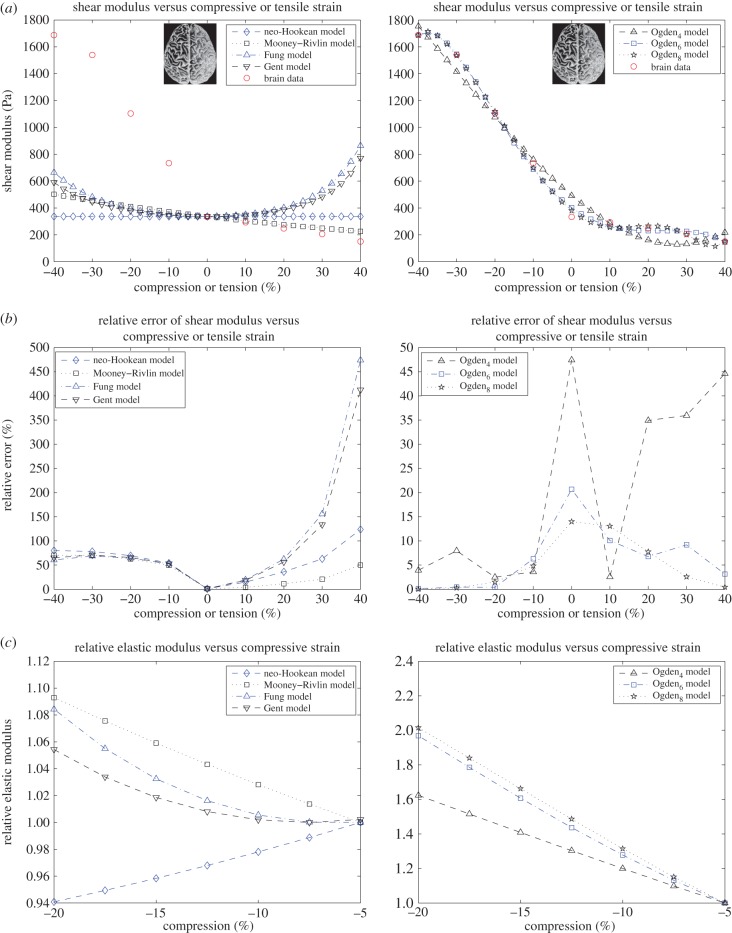
Brain data and models fit. Left: neo-Hookean, Mooney–Rivlin, Fung and Gent models. Right: Ogden_4_, Ogden_6_, Ogden_8_ models. (*a*) Shear modulus *μ* compared with experimental data for brain tissue at 2% shear superposed on up to 40% compression or tension; (*b*) the associated relative errors and (*c*) the elastic modulus *E* normalized to its value at 5% compression.

**Figure 4.**
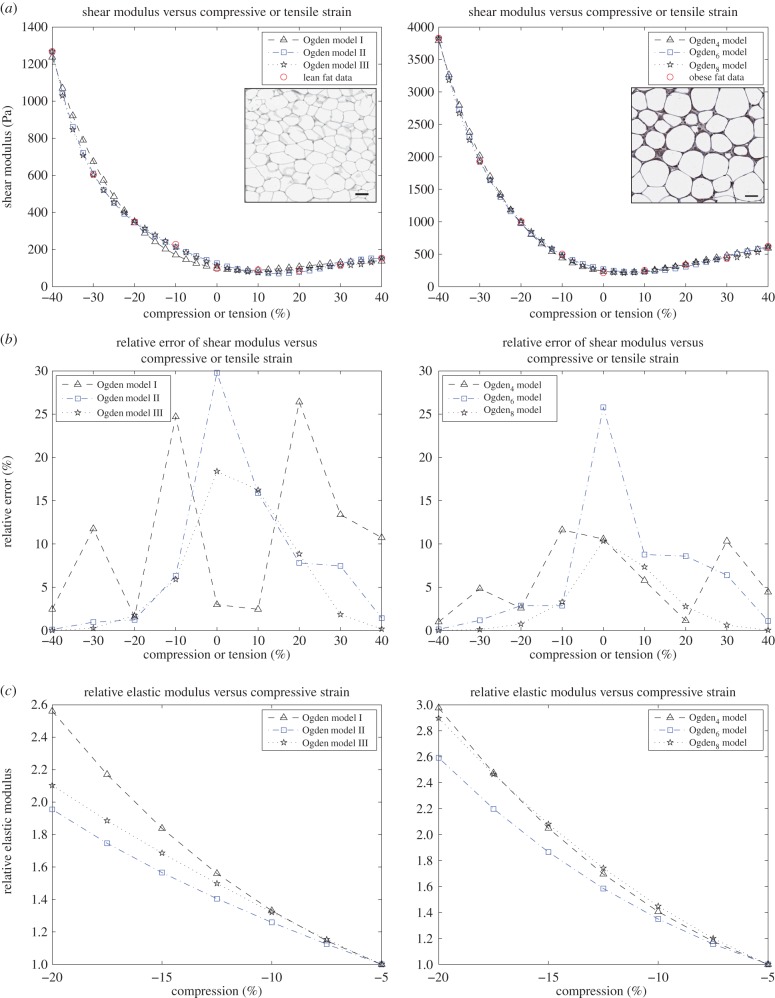
Fat data and models fit. Left: lean fat tissue. Right: obese fat tissue. (*a*) Shear modulus *μ* for Ogden_4_, Ogden_6_, Ogden_8_ models compared with experimental data for fat tissue at 3.5% shear superposed on up to 40% compression or tension; (*b*) the associated relative errors and (*c*) the elastic modulus *E* normalized to its value at 5% compression.

Here, we compare the mechanical performance of the neo-Hookean, Mooney, Fung, Gent and Ogden materials described above when fitted to available experimental data for the shear modulus of brain and fat tissues. According to the experimental measurements, the shear modulus increases strongly under increasing compression, whereas in tension, it remains almost constant or decreases slightly at first, then begins to increase, but much less than in compression. In particular, for brain tissue, the shear modulus is essentially constant up to 10% tensile strain, whereas for lean and obese fat, this modulus appears almost constant up to 30% and 20% tensile strain, respectively. The experimental data for brain and fat tissues are marked by the (red) circles in the plots shown in figures 3*a* and 4*a*, respectively.

The values of the constant parameters for the hyperelastic models fitted to brain and fat data are recorded in tables 2–3 and tables 4–5, respectively. For the neo-Hookean, Mooney–Rivlin, Fung and Gent models, all constant parameters were fitted. For the Ogden models, the non-zero coefficients *C_p_* were fitted, whereas the corresponding exponents *m_p_* were fixed. The fitting of the material parameters was performed using a nonlinear least-squares procedure implemented in Matlab (*lsqnonlin.m*). By this procedure, the following (unconstrained) minimization problem was solved
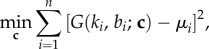
where **c** = (*c*_1_, *c*_2_, … *c_m_*) are the constant material parameters to be identified, (*b_i_*, *μ_i_*) are the pairs of data for the compressive or tensile strain and the shear modulus, respectively, and *G*(*k_i_*, *b_i_*; **c**) = *μ*(*k_i_*, *a_i_*) is the shear modulus defined by (2.6), such that 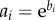 is the stretch parameter, and the shear strain is constant and small, viz. 0.02 for brain and 0.035 for fat tissues, so we set *k_i_* = 0.02*a_i_* and *k_i_* = 0.035*a_i_*, respectively, for all *i* = 1, …, *n*.

**Table 2.** The non-zero parameters for hyperelastic models fitted to shear modulus data for brain tissue at 2% shear superposed on up to 40% compression or tension.

material model	non-zero parameter values
neo-Hookean	*C* = 333.28
Mooney–Rivlin	C_1_ = 0.28, *C*_2_ = 333
Fung	*C* = 166.64, *α* = 2.4974
Gent	*C* = 333.28, *β* = 0.9918

**Table 3.** The non-zero parameters for Ogden models fitted to shear modulus data for brain tissue at 2% shear superposed on up to 40% compression or tension.

material model	non-zero parameter values
Ogden_3_ (brain)	*C*_1_ = −3543, *m*_1_ = 1, *C*_2_ = −2723, *m*_2_ = −1, *C*_3_ = 654, *m*_3_ = 2
Ogden_4_ (brain)	*C*_1_ = −5877, *m*_1_ = 1, *C*_2_ = −5043, *m*_2_ = −1, *C*_3_ = 1161, *m*_3_ = 2, *C*_4_ = 501, *m*_4_ = −2
Ogden_5_ (brain)	*C*_1_ = −34 399, *m*_1_ = 1, *C*_2_ = −18 718, *m*_2_ = −1, *C*_3_ = 14 509, *m*_3_ = 2, *C*_4_ = 2947, *m*_4_ = −2, *C*_5_ = −2349, *m*_5_ = 3
Ogden_6_ (brain)	*C*_1_ = 1189, *m*_1_ = 1, *C*_2_ = 16 855, *m*_2_ = −1, *C*_3_ = 1444, *m*_3_ = 2, *C*_4_ = −10 108, *m*_4_ = −2, *C*_5_ = −458, *m*_5_ = 3, *C*_6_ = 1889, *m*_6_ = −3
Ogden_7_ (brain)	*C*_1_ = −187 150, *m*_1_ = 1, *C*_2_ = −91 970, *m*_2_ = −1, *C*_3_ = 109 290, *m*_3_ = 2, *C*_4_ = 23 200, *m*_4_ = −2, *C*_5_ = −33 290, *m*_5_ = 3, *C*_6_ = −2290, *m*_6_ = −3, *C*_7_ = 4100, *m*_7_ = 4
Ogden_8_ (brain)	*C*_1_ = −639 530, *m*_1_ = 1, *C*_2_ = −544 840, *m*_2_ = −1, *C*_3_ = 322 660, *m*_3_ = 2, *C*_4_ = 237 040, *m*_4_ = −2, *C*_5_ = −88 640, *m*_5_ = 3, *C*_6_ = −57 830, *m*_6_ = −3, *C*_7_ = 10 150, *m*_7_ = 4, *C*_8_ = 6080, *m*_8_ = −4

**Table 4.** The non-zero parameters for Ogden models fitted to shear modulus data for lean fat tissue at 3.5% shear superposed on up to 40% compression or tension.

material model	non-zero parameter values
Ogden_3_ (lean)	*C*_1_ = −3882, *m*_1_ = 1, *C*_2_ = −2113, *m*_2_ = −1, *C*_3_ = 931, *m*_3_ = 2
Ogden_4_ (lean)	*C*_1_ = 2342, *m*_1_ = 1, *C*_2_ = 4083, *m*_2_ = −1, *C*_3_ = −418, *m*_3_ = 2, *C*_4_ = −1337, *m*_4_ = −2
Ogden_5_ (lean)	*C*_1_ = 10 608, *m*_1_ = 1, *C*_2_ = 8054, *m*_2_ = −1, *C*_3_ = −4281, *m*_3_ = 2, *C*_4_ = −2048, *m*_4_ = −2, *C*_5_ = 679, *m*_5_ = 3
Ogden_6_ (lean)	*C*_1_ = −25 361, *m*_1_ = 1, *C*_2_ = −27 961, *m*_2_ = −1, *C*_3_ = 8907, *m*_3_ = 2, *C*_4_ = 11 175, *m*_4_ = −2, *C*_5_ = −1227, *m*_5_ = 3, *C*_6_ = −1914, *m*_6_ = −3
Ogden_7_ (lean)	*C*_1_ = −117 600, *m*_1_ = 1, *C*_2_ = −81 360, *m*_2_ = −1, *C*_3_ = 61 650, *m*_3_ = 2, *C*_4_ = 27 530, *m*_4_ = −2, *C*_5_ = −17 260, *m*_5_ = 3, *C*_6_ = −3970, *m*_6_ = −3, *C*_7_ = 2000, *m*_7_ = 4
Ogden_8_ (lean)	*C*_1_ = −147 280, *m*_1_ = 1, *C*_2_ = −111 070, *m*_2_ = −1, *C*_3_ = 75 640, *m*_3_ = 2, *C*_4_ = 41 560, *m*_4_ = −2, *m*_4_ = −20 890, *m*_5_ = 3, *C*_6_ = −7610, *m*_6_ = −3, *C*_7_ = 2390, *m*_7_ = 4, *C*_8_ = 400, *m*_8_ = −4

**Table 5.** The non-zero parameters for Ogden models fitted to shear modulus data for obese fat tissue at 3.5% shear superposed on up to 40% compression or tension.

material model	non-zero parameter values
Ogden_3_ (obese)	*C*_1_ = −12 779, *m*_1_ = 1, *C*_2_ = −6634, *m*_2_ = −1, *C*_3_ = 3181, *m*_3_ = 2
Ogden_4_ (obese)	*C*_1_ = 6675, *m*_1_ = 1, *C*_2_ = 12 733, *m*_2_ = −1, *C*_3_ = −1036, *m*_3_ = 2, *C*_4_ = −4180, *m*_4_ = −2
Ogden_5_ (obese)	*C*_1_ = 17 032, *m*_1_ = 1, *C*_2_ = 17 708, *m*_2_ = −1, *C*_3_ = −5876, *m*_3_ = 2, *C*_4_ = −5070, *m*_4_ = −2, *C*_5_ = 850, *m*_5_ = 3
Ogden_6_ (obese)	*C*_1_ = −17 287, *m*_1_ = 1, *C*_2_ = −16 654, *m*_2_ = −1, *C*_2_ = 6706, *m*_3_ = 2, *C*_4_ = 7546, *m*_4_ = −2, *C*_5_ = −968, *m*_5_ = 3, *C*_6_ = −1826, *m*_6_ = −3
Ogden_7_ (obese)	*C*_1_ = −294 660, *m*_1_ = 1, *C*_2_ = −177 230, *m*_2_ = −1, *C*_3_ = 165 320, *m*_3_ = 2, *C*_4_ = 56 730, *m*_4_ = −2, *C*_5_ = −49 180, *m*_5_ = 3, *C*_6_ = −8000, *m*_6_ = −3, *C*_7_ = 6010, *m*_7_ = 4
Ogden_8_ (obese)	*C*_1_ = −169 310, *m*_1_ = 1, *C*_2_ = −51 570, *m*_2_ = −1, *C*_3_ = 106 260, *m*_3_ = 2, *C*_4_ = −2630, *m*_4_ = −2, *C*_5_ = −33 880, *m*_5_ = 3, *C*_6_ = 7420, *m*_6_ = −3, *C*_7_ = 4340, *m*_7_ = 4, *C*_8_ = −1690, *m*_8_ = −4

In order to assess the accuracy with which the models capture the mechanical behaviour measured by the experiments, for each model, the relative error of the shear moduli to the given data was also computed, as follows3.1
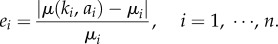


For the neo-Hookean, Mooney, Fung and Gent models with constant parameters as indicated in table 2, the shear moduli at 2% shear combined with up to 40% compression or tension, and their relative errors (3.1) are plotted in figure 3*a*,*b*, respectively. Because the shear strain is small, the shear modulus *μ*_0_ defined by (2.7) is capable of predicting theoretically the corresponding mechanical behaviour of these models under the combined deformation. For these models, we further compute the elastic modulus *E* defined by (2.3), and plot its values normalized to those at 5% compression in figure 3*c*. Numerically

— For the neo-Hookean material, the computed shear modulus *μ* defined by (2.6) is virtually constant, *hence condition* (*ii*) *is valid, but* (*i*) *is not*. For this material also, the relative values of the elastic modulus (2.10) plotted in figure 3*c* decrease when compression increases, *hence condition* (*iii*) *is not valid*. These results are all in agreement with the theoretical findings for the neo-Hookean model.
— For the Mooney–Rivlin material, the shear modulus *μ* increases as compression increases and decreases as tension increases, *thus conditions* (*i*) *and* (*ii*) *are both satisfied*. From the relative values of the elastic modulus plotted in figure 3*c*, we also see that, for this material, the elastic modulus (2.11) increases under increasing compression, *i.e. condition* (*iii*) *is also satisfied*. These results are again in agreement with the theoretical findings for the Mooney model. Unfortunately, the numerical values of the shear modulus attained by this model are much smaller than those required by the experimental results for brain tissue, as shown by the large relative error estimates, hence Mooney materials, which have proved excellent in describing elastomers and other materials with entropic elasticity, are inadequate for the modelling of this tissue.
— For the Fung and the Gent materials, the respective shear moduli *μ* increase as compression increases, i.e. *condition* (*i*) *is satisfied*, but because they also increase almost as fast in tension as in compression, *condition* (*ii*) *is not satisfied*. Moreover, the corresponding relative errors increase rapidly as either compression or tension increases, hence, these materials do not capture the required physical behaviour in either of these deformations. For these models also, the monotonicity of the associated elastic modulus (2.12) and (2.13) changes, albeit slowly, so that the computed modulus remains almost constant before it increases as compression increases. *Hence*, *condition* (*iii*) *is, in fact, satisfied numerically*.

As the neo-Hookean, Mooney, Fung and Gent models fail to agree with the experimental results for brain tissue under combined stretch and shear, and similar results are shown to hold experimentally for adipose tissue, we illustrate numerically the behaviour of these material models in rapport to the brain data, but take these models no farther when modelling fat tissues (table 6).

— For brain and fat tissues, we further determine six different Ogden-type models, with the associated constant parameters recorded in tables 3–5. In tables 3–5, the Ogden*_N_* models have *N* non-zero coefficients *C_p_*, whereas the associated exponents *m_p_* are fixed. For these models, *conditions* (*i*) *and* (*ii*) *are both valid*. See also figures 3*a* and 4*a*. The relative errors recorded in tables 7–9 further suggest that Ogden_7_ and Ogden_8_ are the most successful in approximating the experimental data. Obviously, these last models contain a large number of parameters and are likely to over-fit the data. The purpose of including these models is to demonstrate that such a family of models is adequate to capture the mechanical responses of the biological tissues under investigation. See also figures 3*b* and 4*b*. From the associated relative elastic modulus plotted in figures 3*c* and 4*c*, we also see that this modulus increases under increasing compression, *hence condition* (*iii*) *is also valid*. For all models, smaller relative values for the elastic modulus may be obtained when this modulus is defined by (2.4).

**Table 6.** Relative errors of the shear modulus for hyperelastic models fitted to brain data.

compression or tension (%)	relative error (%)			
	neo-Hookean (brain)	Mooney (brain)	Fung (brain)	Gent (brain)
−40.00	80.05	70.25	60.61	50.18
−30.00	78.12	70.47	68.71	53.01
−20.00	69.50	62.75	64.51	51.97
−10.00	54.16	49.34	52.41	32.97
0.00	1.00	1.00	1.05	0.05
10.00	14.68	3.78	19.40	6.40
20.00	36.15	11.49	62.31	7.56
30.00	62.86	20.69	155.88	3.73
40.00	123.73	50.03	474.16	51.08

**Table 7.** Relative errors of the shear modulus for Ogden models fitted to brain data.

compression or tension (%)	relative error (%)					
	Ogden_3_ (brain)	Ogden_4_ (brain)	Ogden_5_ (brain)	Ogden_6_ (brain)	Ogden_7_ (brain)	Ogden_8_ (brain)
−40.00	7.03	3.93	0.67	0.12	0.07	0.03
−30.00	9.81	7.96	1.98	0.44	0.57	0.28
−20.00	6.67	2.49	1.74	0.42	2.45	1.49
−10.00	0.35	3.61	2.49	6.31	6.24	4.83
0.00	47.87	47.44	20.59	20.67	13.99	14.00
10.00	7.22	2.57	18.80	10.07	9.85	13.05
20.00	19.13	34.92	11.50	6.76	4.27	7.75
30.00	26.09	35.97	22.83	9.19	0.98	2.58
40.00	19.20	44.63	9.27	3.14	0.10	0.42

**Table 8.** Relative errors of the shear modulus for Ogden models fitted to lean fat data.

compression or tension (%)	relative error (%)					
	Ogden_3_ (lean)	Ogden_4_ (lean)	Ogden_5_ (lean)	Ogden_6_ (lean)	Ogden_7_ (lean)	Ogden_8_ (lean)
−40.00	13.57	2.46	1.19	0.11	0.02	0.01
−30.00	24.42	11.75	7.29	0.97	0.31	0.26
−20.00	34.01	1.57	5.49	1.22	1.95	1.76
−10.00	9.86	24.70	18.93	6.31	6.20	5.90
0.00	0.96	2.98	30.03	29.80	18.40	18.39
10.00	87.37	2.44	12.90	15.87	15.55	16.23
20.00	97.84	26.41	6.41	7.82	8.15	8.84
30.00	34.75	13.39	17.74	7.46	1.65	1.85
40.00	56.19	10.73	4.70	1.42	0.14	0.17

**Table 9.** Relative errors of the shear modulus for Ogden models fitted to obese fat data.

compression or tension (%)	relative error (%)					
	Ogden_3_ (obese)	Ogden_4_ (obese)	Ogden_5_ (obese)	Ogden_6_ (obese)	Ogden_7_ (obese)	Ogden_8_ (obese)
−40.00	12.50	1.00	0.47	0.13	0.00	0.00
−30.00	17.25	4.82	3.07	1.18	0.03	0.10
−20.00	36.17	2.58	4.29	2.87	0.45	0.75
−10.00	37.45	11.62	8.34	2.87	2.72	3.30
0.00	4.99	10.57	25.89	25.78	10.30	10.33
10.00	102.75	5.77	1.25	8.78	8.43	7.35
20.00	96.61	1.13	5.18	8.59	3.49	2.78
30.00	29.33	10.34	0.05	6.39	0.82	0.61
40.00	47.42	4.44	0.36	1.09	0.07	0.05

As explained above, for brain and fat tissues, it was observed experimentally that the shear modulus increases sharply under increasing compressive strain, but not under tensile strain, whereas the apparent elastic modulus increases or remains almost constant when compressive strain increases. The macroscopic, centimetre-scale, samples are heterogeneous on a smaller length scale, because they are a mix of grey and white matter with boundaries between, but this heterogeneity does not dominate the rheological response, because grey and white matter do not differ strongly in stiffness, and the macroscopic viscoelastic response does not depend on precisely how the sample is cut or how large it is. In addition, a characteristic of these tissues is that they exhibit a predominantly isotropic incompressible behaviour. Here, we compare the behaviours of several nonlinear hyperelastic models, both theoretically and numerically, and test our results against available experimental data. Our analysis shows that neo-Hookean, Mooney–Rivlin, Fung and Gent models, which have been successfully employed to date in the modelling of rubber and of other man-made or natural materials, are inadequate to model brain and fat tissues. Instead, for these tissues, Ogden models, with four, six and eight coefficients, respectively, are found which are in excellent agreement with the experiments. The newly identified models can be easily implemented in finite-element codes.

## 4. Conclusion

Biological tissues offer a great diversity of mechanical responses when subject to loads. Often, such behaviours appear counterintuitive as our intuition has been forged by centuries of studies of engineering material often treated in the limit of small strains. It is then tempting to conclude that classical continuum mechanics is not suitable for modelling biological materials. However, aside from the very few classical models used indiscriminately for both rubbers and biological tissues, there is a vast pool of potential models that have yet to be explored, understood and classified.

Our approach when confronted with a new constitutive phenomenon consists of two steps: first, based on experimental evidence, classify qualitative responses that a model ought to reproduce by an analytical study of relevant deformations. Second, for the models that pass the first sift, find suitable candidates in quantitative agreement with the data. As presented here, this approach was successful for the analysis of the response of brain and fat tissues, generating in the process models that are capable of predicting some of the key elastic properties underpinning the extraordinary mechanical performance of these tissues, and which can be integrated into large-scale computational framework. Far for claiming that the models presented here are universal models for brain and fat tissues, we demonstrate that a systematic approach in the framework of nonlinear elasticity based on experimental data provides phenomenological models that can be used to explore the large-scale response of tissues and organs. We have listed several models that fit the data increasingly well at the expense of an increase in the number of parameters. We leave it to the practitioners to decide, based on the problem at hand and the range of deformation being studied, which model to use.

Our enquiry also suggests that the microscopic processes that generate macroscopic elastic responses in these tissues are different to the ones found in other soft tissues or elastomers. Clearly, there is a need for better understanding of the mechanics of very soft tissues, particularly of the brain, which is currently under intense study by researchers in both biophysics and computational mechanics, and adipose tissue, which is a growing area of investigation in clinical research.

## Supplementary Material

brain-fat-shear-modulus-data

## Supplementary Material

brain-strain-sweep

## Supplementary Material

fat-strain-sweep

## Supplementary Material

time-sweeps

## Figures and Tables

**Figure 5.**
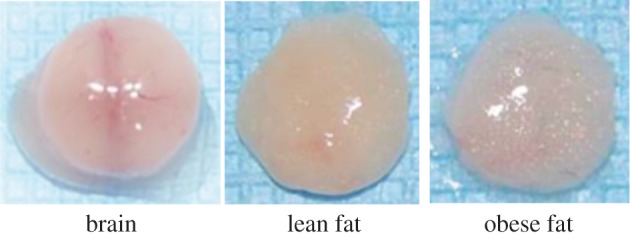
Typical samples of brain, obese, and lean fat tissues are cut using an 8 mm diameter stainless steel punch. Major brain structures, such as cerebellum and olfactory bulb, are removed. The samples did not exhibit any anisotropic characteristics.

**Figure 6.**
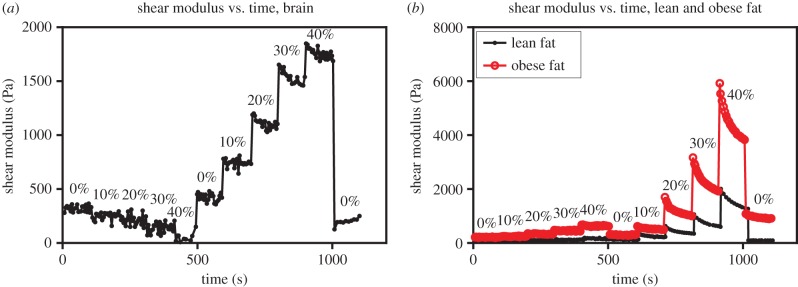
(*a*) Experimental data for brain. Shear modulus was measured over time and with increasing tension/compression (0% to 40%). *G*′ relaxes to equilibrium after 100 seconds. Recoverable deformation was observed when the compressed sample was returned to its initial height and the shear modulus *G*′ returned to its uncompressed value. (*b*) Experimental data for lean and obese fat. Shear modulus was measured over time and with increasing tension/compression (0% to 40%). *G*′ does not relax completely to equilibrium after 100 seconds, particularly for compression levels of 30% and above. For lean fat, recoverable deformation was observed when the compressed sample was returned to its initial height and the shear modulus *G*′ returned to its uncompressed value. Obese fat largely recovers, but residual stresses appear to remain after 40% compression.

**Figure 7.**
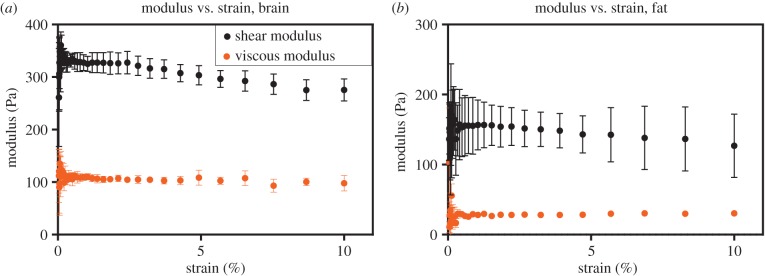
(*a*) Shear modulus of brain with increasing shear strains. The linear viscoelastic region was determined to be approximately 0.15-2.5% strain. (*b*) Shear modulus of lean fat with increasing shear strains. The linear viscoelastic region was determined to be 0.15-4% strain.

## Data accessibility

The datasets supporting this article have been uploaded as part of the electronic supplementary material.

## Appendix A. Experimental method

Brain and lean adipose tissues were harvested from adult male wild-type C57BL/6 mice and obese adipose tissue was collected from genetically obese ob/ob mice (The Jackson Laboratory, Bar Harbor, ME). Fresh tissues were stored in Dulbecco's modified Eagle's medium (DMEM, Gibco, Grand Island, NY) and tested within a maximum of 3 h after sacrifice. Macroscopic rheometry using a Rheometrics fluids spectrometer III strain-controlled rheometer (Rheometrics, Piscataway, NY) fitted with 8 mm diameter parallel plates was used to measure the viscoelasticity of tissue samples using methods previously described in [4]. During testing, tissues were kept hydrated by surrounding the sample with phosphate-buffered saline (PBS). Control experiments showed that the use of DMEM and PBS increased tissue weight only slightly—on average 4% for brain, 2% for fat.

Test samples were cut into disc-shaped samples using an 8 mm diameter stainless steel punch, maintaining the cerebral hemispheres, but removing the cerebellum, olfactory bulb, pons and medulla (figure 5). Note that an 8 mm diameter disc in the brain will necessarily contain a mixture of different tissues and structures. However, structures such as ventricles and vessels constitute a small percentage of the volume (2%) and are not arranged in a highly oriented manner. The bulk of the tissues consists of cells packed together and surrounded by soft matrices, which in the brain are flexible polysaccharides and so very likely to be close to isotropic (as found repeatedly in other experiments [1]). Because the length scale of the individual tissue components is much smaller than the length scale of the bulk tissue, the protocol provides an average mechanical response suitable for comparison with continuum models. If swelling occurs and we assume that it deforms the sample equally in all directions, a typical 4% increase in volume yields 1% increase in height. A 1% change in height and the effect on *G*′ are relatively small compared with the deformations the samples are subjected to during measurement.

To avoid sample slippage during shear deformation and to perform uniaxial extension, fibrin gel was used to glue the sample to the rheometer plate. Fibrin gel was prepared by mixing equal volumes of 28 mg ml^−1^ salmon fibrinogen solution with 125 U ml^−1^ of thrombin (Sea Run Holdings, Freeport, ME) directly on the lower rheometer plate, and the sample was immediately positioned. Subsequently, a thin layer of fibrin gel was pipetted onto the upper surface of the tissue, and the top plate was lowered until a positive normal force (1 g) was measured by a force transducer. Control experiments showed that addition of fibrin glue did not affect the viscoelastic properties of the tissue samples.

The dynamic shear storage modulus *G*′ was measured as a function of time (brain: 2% oscillatory shear strain, 2 rad s^−1^ frequency; fat: 3.5% shear strain, 2.5 rad s^−1^ frequency) and increasing tensile/compressive strain (0–40%; figure 5). For both brain [4] and lean fat, recoverable deformation was observed when the compressed sample was returned to its initial height and the shear modulus *G*′ returned to its uncompressed value (figure 6). Obese fat largely recovers, but residual stresses appear to remain after 40% compression. This may be indicative of tissue damage at higher levels of compression, but does not negate the compression-stiffening phenomenon observed. During each incremental compression, *G*′ demonstrated a relaxation response. Therefore, for the purposes of model fitting, *G*′ values after 100 s of relaxation were used. In tension, the following correction was applied to account for the decrease in cross-sectional area during testing under the assumption that volume is conserved,  where *G*′ is the storage modulus and *λ* is the axial strain. To determine the linear viscoelastic region for each tissue, the shear moduli *G*′ of brain and lean fat were measured with respect to increasing shear strain up to 10% (*n* = 3 each, figure 7). The experimentally measurable linear viscoelastic region for brain and fat were determined to be 0.15–2.5% and 0.15–4% strain, respectively.
